# Spatial-temporal epidemiology of human *Salmonella* Enteritidis infections with major phage types (PTs 1, 4, 5b, 8, 13, and 13a) in Ontario, Canada, 2008–2009

**DOI:** 10.1186/s12889-015-2592-6

**Published:** 2015-12-17

**Authors:** Csaba Varga, David L. Pearl, Scott A. McEwen, Jan M. Sargeant, Frank Pollari, Michele T. Guerin

**Affiliations:** Department of Population Medicine, Ontario Veterinary College, University of Guelph, Guelph, ON N1G 2W1 Canada; Ontario Ministry of Agriculture, Food and Rural Affairs, Guelph, ON N1G 4Y2 Canada; Centre for Public Health and Zoonoses, Ontario Veterinary College, University of Guelph, Guelph, ON N1G 2W1 Canada; Centre for Foodborne, Environmental and Zoonotic Infectious Diseases, Public Health Agency of Canada, Guelph, ON N1H 8J1 Canada

**Keywords:** Phage type, Surveillance, Retrospective, Travel, Restaurant, Scan statistic, *Salmonella* Enteritidis, Canada

## Abstract

**Background:**

In Ontario and Canada, the incidence of human *Salmonella enterica* serotype Enteritidis (*S.* Enteritidis) infections have increased steadily during the last decade. Our study evaluated the spatial and temporal epidemiology of the major phage types (**PTs**) of *S.* Enteritidis infections to aid public health practitioners design effective prevention and control programs.

**Methods:**

Data on *S.* Enteritidis infections between January 1, 2008 and December 31, 2009 were obtained from Ontario’s disease surveillance system. *Salmonella* Enteritidis infections with major phage types were classified by their annual health region-level incidence rates (**IRs**), monthly IRs, clinical symptoms, and exposure settings. A scan statistic was employed to detect retrospective phage type-specific spatial, temporal, and space-time clusters of *S.* Enteritidis infections. Space-time cluster cases’ exposure settings were evaluated to identify common exposures.

**Results:**

1,336 cases were available for analysis. The six most frequently reported *S.* Enteritidis PTs were 8 (*n* = 398), 13a (*n* = 218), 13 (*n* = 198), 1 (*n* = 132), 5b (*n* = 83), and 4 (*n* = 76). Reported rates of *S.* Enteritidis infections with major phage types varied by health region and month. International travel and unknown exposure settings were the most frequently reported settings for PT 5b, 4, and 1 cases, whereas unknown exposure setting, private home, food premise, and international travel were the most frequently reported settings for PT 8, 13, and 13a cases.

Diarrhea, abdominal pain, and fever were the most commonly reported clinical symptoms. A number of phage type-specific spatial, temporal, and space-time clusters were identified. Space-time clusters of PTs 1, 4, and 5b occurred mainly during the winter and spring months in the North West, North East, Eastern, Central East, and Central West regions. Space-time clusters of PTs 13 and 13a occurred at different times of the year in the Toronto region. Space-time clusters of PT 8 occurred at different times of the year in the North West and South West regions.

**Conclusions:**

Phage type-specific differences in exposure settings, and spatial-temporal clustering of *S.* Enteritidis infections were demonstrated that might guide public health surveillance of disease outbreaks. Our study methodology could be applied to other foodborne disease surveillance data to detect retrospective high disease rate clusters, which could aid public health authorities in developing effective prevention and control programs.

**Electronic supplementary material:**

The online version of this article (doi:10.1186/s12889-015-2592-6) contains supplementary material, which is available to authorized users.

## Background

Salmonellosis is a major foodborne bacterial infection that continuously poses a significant human health burden worldwide [1]. In Canada, salmonellosis is the main cause of hospitalization and death among domestically acquired foodborne infections [2], causing an estimated 87,510 illnesses annually [3]. In the last decade, *Salmonella enterica* serotype Enteritidis (***S.*Enteritidis**) became the top serovar among the non-typhoidal salmonellae in Canada [4], the United States of America (**US**) [5, 6], and the European Union [7].

Currently in Canada, the predominant *S.* Enteritidis phage types (**PTs**) among human cases are PT 8, 13a, 13, 1, 4, and 5b [4]. Between 2006 and 2010, Canadian integrated surveillance systems identified the emergence of PT 13a and an increase in the number of cases of PT 8 [4].

Several research studies conducted in North America have evaluated phage type-specific risk factors for *S.* Enteritidis infections in humans. In Ontario, Canada, researchers demonstrated that cases with PT 8 were more likely to have had contact with dogs compared to cases with other phage types [8]. In British Columbia, Canada, a concurrent increase in the incidence of *S.* Enteritidis infections with PT 8 in humans and the prevalence of PT 8 in poultry was observed between 2007 and 2010 [9]. The researchers demonstrated increased odds of infection with PT 8 in human cases who consumed illegally-sourced ungraded eggs compared to controls [9]. In Alberta, Canada, an outbreak of PT 8, 13, and atypical PTs was linked to the consumption of food products purchased from mobile lunch trucks that were contaminated by illegally-obtained eggs and/or by infected food handlers [10]. In the US, PT 8 cases were more likely to have consumed chicken or be the owner of a lizard than controls, whereas PT 13 cases were more likely to have eaten undercooked eggs in their home than controls [11].

In Ontario and Canada, an increase in the reported number of human *S.* Enteritidis cases was observed during the last decade [12, 13]. Current Ontario studies revealed that the majority of *S.* Enteritidis cases with PT 1, 4, or 6a were international travel-related, whereas cases with PT 8, 13, or 13a were mainly acquired domestically [14, 15]. These studies provided valuable information on the seasonality and exposure locations of *S.* Enteritidis cases, although they lacked information on cases’ geographical distribution and spatial-temporal clustering. Identifying areas with high rates of reported *S.* Enteritidis cases can be useful for targeting prevention and control programs [12, 16].

There have been a limited number of studies that evaluated foodborne disease surveillance data by incorporating geographical information system (GIS) data, spatial-temporal scan statistic results, exposure setting information, and clinical syndrome history. Scan statistics have been effectively used to evaluate clustering and transmission dynamics of pandemic influenza A (H1N1) in Hong Kong, China [17], to detect *Escherichia coli* O157:H7 outbreaks involving common molecular subtypes in Alberta, Canada [18], to identify the location of high and low rate areas of campylobacteriosis incidence in Manitoba, Canada [19], to identify high incidence clusters of tuberculosis in Linyi City, China [20], and to find childhood cancer clusters in Alberta, Canada [21].

This study assesses the spatial and temporal epidemiology of the phage types of *S.* Enteritidis that predominate in Ontario health regions by: 1) estimating phage type-specific health region-level incidence rates (**IRs**); 2) estimating phage type-specific monthly IRs; 3) describing phage type-specific exposure settings and clinical symptoms; 4) detecting phage-type-specific spatial, temporal, and space-time clusters of cases; and 5) examining the exposure settings of cases identified within space-time clusters. The results of this study are expected to assist public health officials with the development of disease prevention programs within the province.

## Methods

### Study setting and data sources

**Fig. 1:**
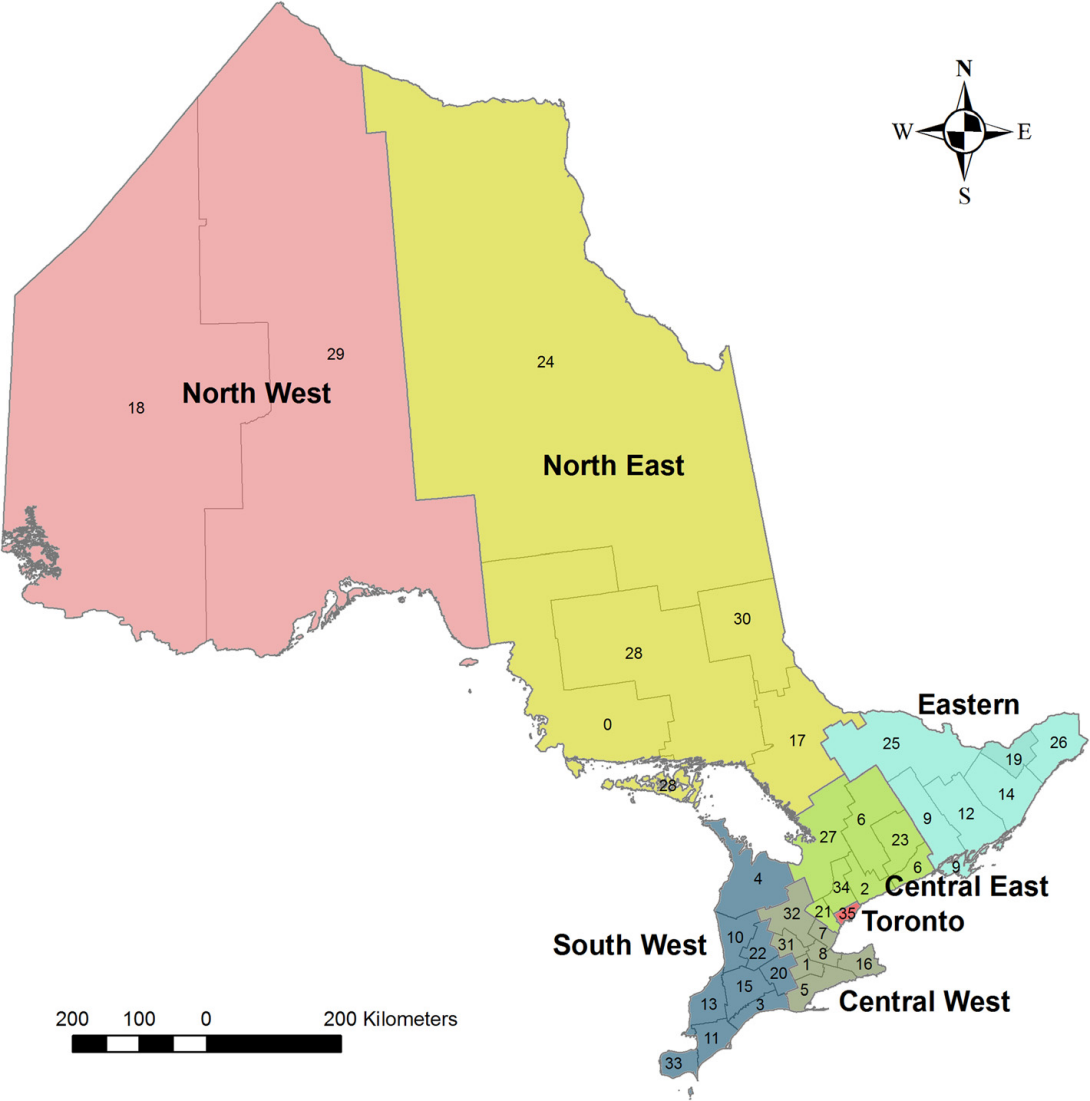
Health regions in Ontario, Canada. The names and population estimates for the public health units (indicated by labels 0 through 35), are presented in Additional file 1: Legend 1

Our study was conducted in Ontario, Canada. In 2009, an estimated 13 million people lived in Ontario, accounting for 39 % of Canada’s total population [22]. There are 36 public health units (**PHUs**) in Ontario that are mandated by the provincial ministry of health to administer health promotion and disease prevention programs [23]. These PHUs are grouped into seven planning regions, which were used for the purposes of our study (Fig. 1; Additional file 1: Legend 1).

Salmonellosis is a reportable disease under provincial legislation [23], and is diagnosed by public health, hospital, and private laboratories after isolation of *Salmonella* spp. (excluding *Salmonella* Typhi or Paratyphi) from stool (the majority of samples), rectal swabs, urine, blood, or any other sterile site [24]. All *Salmonella* isolates are sent to the Public Health Ontario Laboratories-Toronto for confirmation and serotyping using serological confirmation of compatible somatic and flagellar antigens (Kauffmann-White classification) [25]. All isolates serotyped as *S.* Enteritidis are sent to the National Microbiology Laboratory in Winnipeg, Manitoba for phage typing using techniques defined by Ward and colleagues [26].

Staff at each PHU in Ontario must follow up with every *S.* Enteritidis case to identify exposure settings during the illness incubation period and the clinical symptoms during illness. Case investigation records must be reported to the Ontario Ministry of Health and Long-Term Care (**MOHLTC**) through the integrated Public Health Information System (**iPHIS**). Each PHU has its own case follow-up protocol, and currently there is no standardized follow-up form or set timeline for initial case contact. The exposure setting information is based on what the case reported and was considered significant by the investigator. Exposure settings in the surveillance database were categorized as: international travel (i.e., travelled outside of Canada), private home, food premises (e.g., restaurant, grocery store, bakery, deli, caterer, mobile food premise), other (e.g., institution, hospital, farm, petting zoo, child care centre), or unknown (if the only exposure reported was “unknown”). Cases without exposure setting details were excluded from the exposure setting analysis. When more than one exposure setting was reported, the primary exposure was included in our analysis. Secondary exposure was only considered when the primary exposure was reported as “unknown”.

### Statistical analysis

#### Data management

**Fig. 2:**
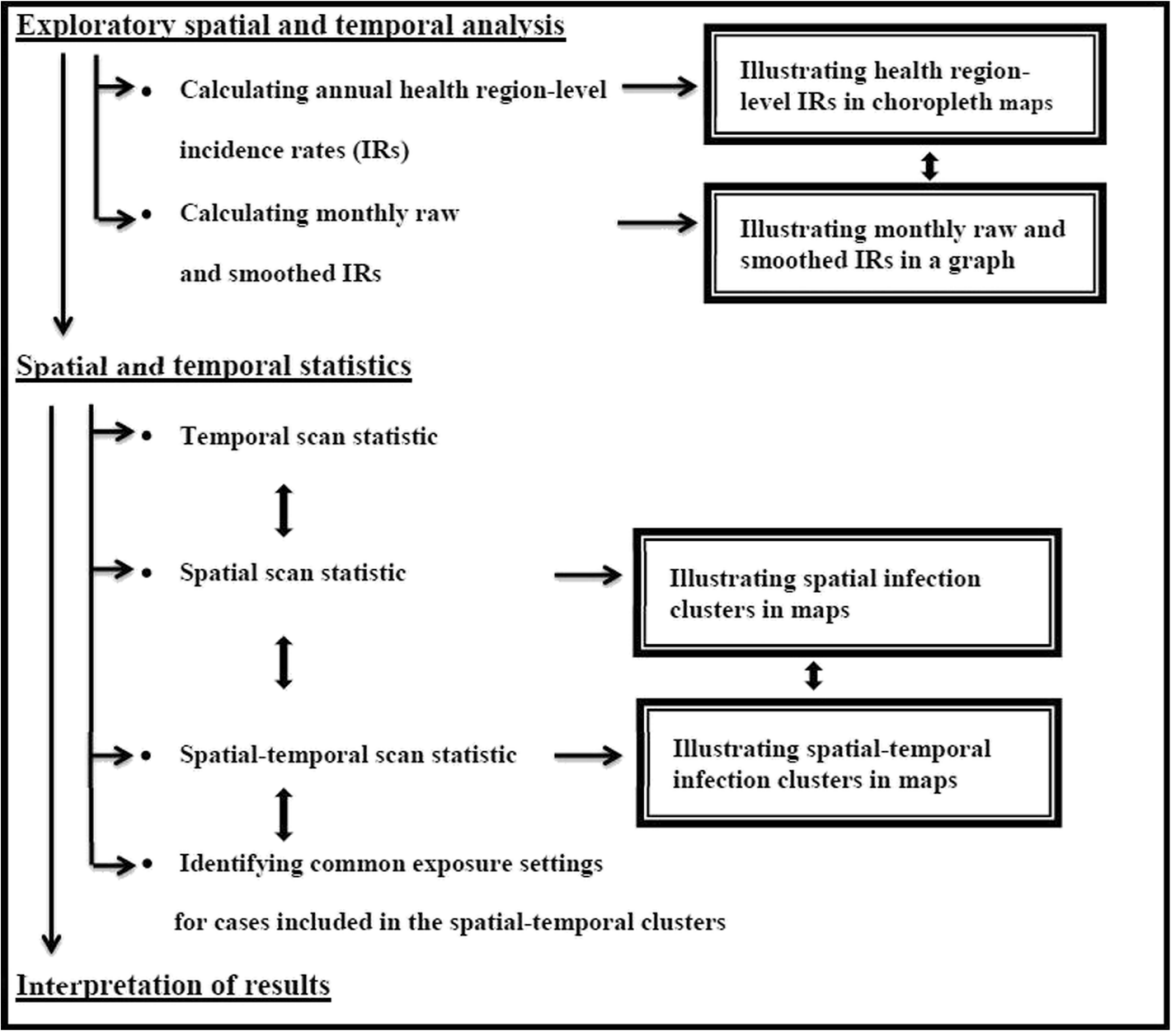
Flow chart outlining the analytical steps used to evaluate *Salmonella* Enteritidis cases with major phage types

Data pertaining to the *S.* Enteritidis cases’ phage type, age, sex, reporting PHU, date of illness onset, exposure setting, and clinical symptoms were acquired from the iPHIS passive surveillance database. Data were entered into a spreadsheet program (Microsoft Excel 2010, Microsoft Corporation, Redmond, WA, US), reviewed for missing values, and subsequently imported into STATA Intercooled statistical software, version 10.1 (Stata Corporation, College Station, TX, US) for descriptive statistical analysis. Data were available from January 1, 2007 to December 31, 2009; however, due to the large amount of missing phage type information in 2007, all cases from 2007 were excluded from the analyses. Therefore, we evaluated all *S.* Enteritidis cases that were captured within the iPHIS database between January 1, 2008 and December 31, 2009. The frequency of *Salmonella* Enteritidis phage types was calculated, the most commonly reported phage types were identified (>5 % of the total number of *S.* Enteritidis cases that were phage typed during the 2-year study period), and the spatial and temporal epidemiology of these phage types were assessed by following several analytical steps, which are outlined in Fig. 2 and described in detail below. Cartographical boundary files and population estimates for each health region were acquired from Statistics Canada [27].

### Phage type-specific incidence rates

Health region-level IRs for the six most commonly reported *S.* Enteritidis phage type cases were calculated by dividing the number of cases in a health region with the phage type during the 2-year study period by the population estimate for the health region for the 2-year study period. Health region-level phage-type specific IRs were illustrated in choropleth maps using ArcGIS 10 software (ESRI Inc., Redlands, CA, US).

For the entire province, monthly IRs for the six most frequent phage types were calculated by dividing the number of cases in a month with the phage type by the monthly population estimate. Smoothed IRs based on a simple 3-month moving average were calculated in Microsoft Excel 2010 and plotted together with the monthly raw IRs.

### Scan statistic

Individual models were built for the 2-year study period for the six most frequent phage types in Ontario. Scan statistics using discrete Poisson models [28] in SaTScan software version 9.0 [29] were conducted to identify purely spatial, purely temporal, and space-time clusters of *S.* Enteritidis cases. The assumption of the Poisson model is that the number of cases in each health region are Poisson-distributed, based on a known underlying population at risk [28, 30]. Cartesian coordinates of latitude and longitude for each health region centroid were calculated in ArcGIS 10. The smallest spatial and temporal unit was the centroid of a health region and the month of disease onset, respectively. Only high rate clusters were investigated. Secondary clusters were reported if they did not overlap in space with the primary cluster. The scan statistic uses a circular scanning window in space, an interval in time, and a cylinder with a circular spatial base and height corresponding to time in space-time [28, 30]. The scanning window of variable radii gradually moves through time and/or space comparing the rate of cases inside the scanning window to outside the window. When the rate inside the scanning window compared to outside is higher than expected by random chance alone, a high rate cluster is identified. A relative risk and a *p*-value obtained through Monte Carlo hypothesis testing using 999 replications were estimated for each cluster [31]. A *p*-value ≤0.05 was considered to be significant. The maximum scanning window size was set to include up to 50 % of the population at risk and up to 50 % of study period [28, 30]. Analyses were adjusted for age (0–9, 10–24, 25–34, 35–49, ≥ 50 years) and sex covariates [28]. Statistically significant spatial and space-time clusters were illustrated using a map with health region boundaries in ArcGIS 10. The exposure settings of cases that were part of statistically significant space-time clusters were obtained from iPHIS and examined to assess if a common exposure explained the clustering of cases in space-time.

## Results

A total of 1,364 *S.* Enteritidis cases were recorded in the iPHIS database during the study period; of these, 28 cases were missing phage type information, leaving 1,336 cases (97.9 %) available for analysis. The most commonly reported phage types were PT 8 (*n* = 398), PT 13a (*n* = 218), PT 13 (*n* = 198), PT 1 (*n* = 132), PT 5b (*n* = 83), and PT 4 (*n* = 76), which together accounted for 82.7 % of all *S.* Enteritidis cases in Ontario with known phage types during the study period (Table 1). One PT 8 case was excluded from the scan statistics because of missing sex information. No outbreaks (e.g., two or more cases linked epidemiologically) were declared by the MOHLTC during the study period.

**Table 1:** Frequency of *Salmonella* Enteritidis cases with different phage types in Ontario, Canada, 2008-2009 (*n* = 1,336)

Phage type	n (n/N %)	Phage type	n (n/N%)
PT 8	398 (29.8)	PT 1a	11 (0.8)
PT 13a	218 (16.3)	PT 21	11 (0.8)
PT 13	198 (14.8)	PT 1b	10 (0.7)
PT 1	132 (9.9)	PT 14b	10 (0.7)
PT 5b	83 (6.2)	PT 22	9 (0.7)
PT 4	76 (5.7)	PT 23	8 (0.6)
PT 6a	49 (3.7)	PT 19	7 (0.5)
atypical	33 (2.5)	PT 51	6 (0.4)
PT 6	20 (1.5)	other	57 (4.3)

*n* = number of *S.* Enteritidis cases with the phage type. *N* = total number of *S.* Enteritidis cases that were phage typed during the 2-year study period = 1,336

### Health region-level incidence rates

**Fig. 3:**
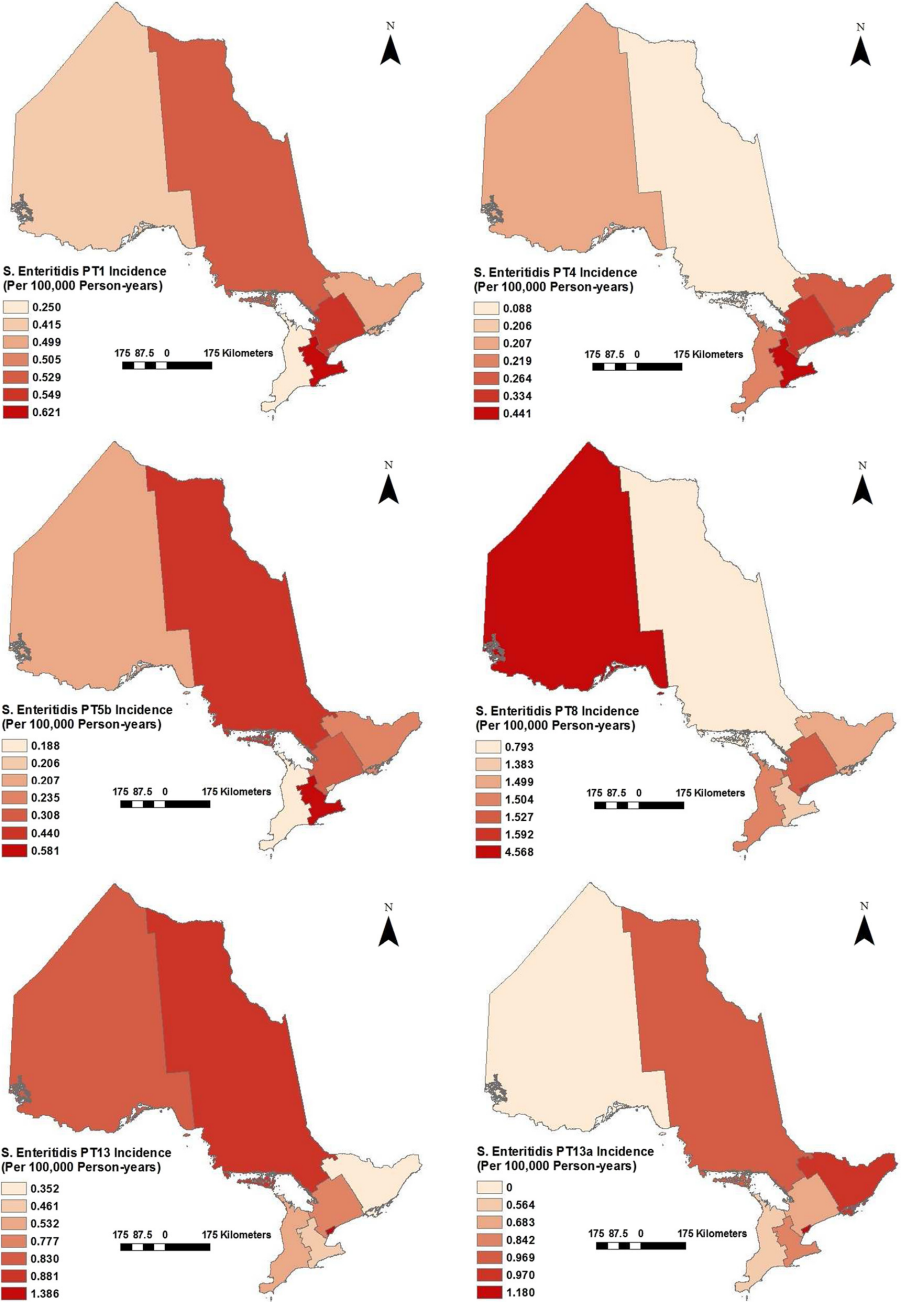
Health region-level raw incidence rates of *Salmonella* Enteritidis cases with major phage types in Ontario, Canada, 2008–2009

Figure 3 illustrates the annual health region-level IRs of *S.* Enteritidis infections per 100,000 person-years for the six most frequent phage types in Ontario, and described below. For PT 1, the IR ranged from 0.25 to 0.62 units (mean = 0.48), with the highest IRs observed in the Central West and Central East regions. For PT 4, the IR ranged from 0.09 to 0.44 units (mean = 0.25), with the highest IRs observed in the Central West and Central East regions. For PT 5b, the IR ranged from 0.19 to 0.58 units (mean = 0.31), with the highest IRs observed in the Central West and North East regions. For PT 8, the IR ranged from 0.79 to 4.57 units (mean = 1.84), with the highest IRs observed in the North West and Toronto regions. For PT 13, the IR ranged from 0.35 to 1.39 units (mean = 0.75), with the highest IRs observed in the Toronto and North West regions. For PT 13a, the IR ranged from 0 to 1.18 units (mean = 0.74), with the highest IRs observed in the Toronto and Eastern regions.

### Monthly raw and smoothed incidence rates

**Fig. 4:**
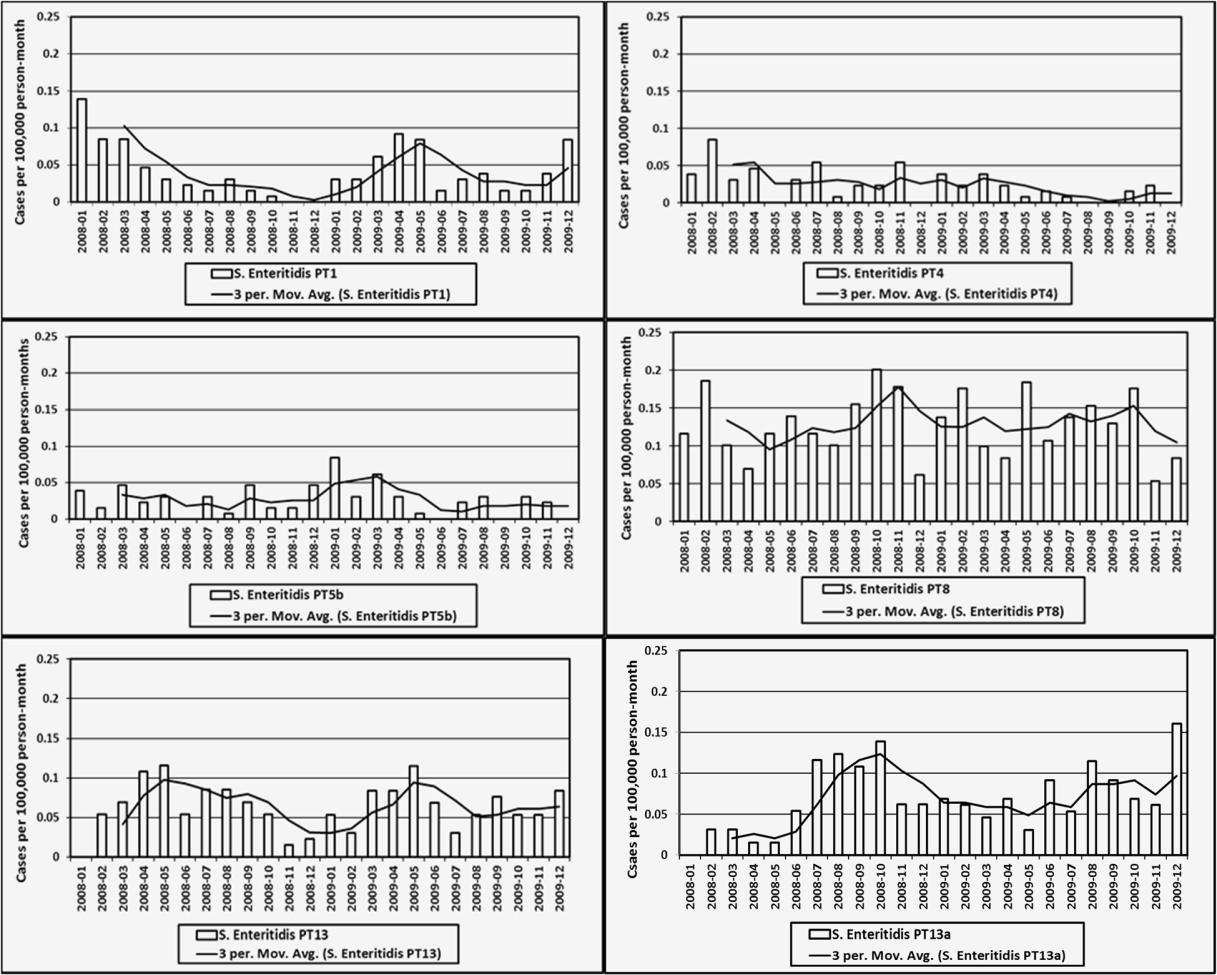
Monthly raw and smoothed incidence rates of *Salmonella* Enteritidis cases with major phage types in Ontario. Smoothed IRs were based on a 3-month simple rolling average

Time-series of raw and smoothed IRs of *S.* Enteritidis infections per 100,000 person-months for the six most frequent phage types in Ontario are illustrated in Fig. 4, and are described below. The monthly IR ranged from 0 to 0.14 units (mean = 0.04) for PT 1, 0 to 0.09 units (mean = 0.02) for PT 4, 0 to 0.08 units (mean = 0.03) for PT 5b, 0.05 to 0.20 units (mean = 0.13) for PT 8, 0 to 0.12 units (mean = 0.06) for PT 13, and 0 to 0.16 units (mean = 0.07) for PT 13a.

Visually assessing the smoothed trend lines, a number of patterns were observed (Fig. 4). For PT 1, there were steep up slopes and gradual down slopes, with peaks occurring in January 2008, May 2009, and December 2009. For PT 4, there were three small peaks, which occurred in February 2008, November 2008, and March 2009. For PT 5b, there was one high peak in January 2009. For PT 8, there were monthly variations with five peaks, which occurred in February 2008, November 2008, February 2009, May 2009, and October 2009. For PT 13, there were two peaks, which occurred in May 2008 and May 2009. For PT 13a, there was a high plateau between August 2008 and October 2008, a small plateau between August 2009 and October 2009, and a small peak in December 2009.

### Clinical symptoms

Of the 1,336 *S.* Enteritidis cases with known phage types, 1,123 cases (84.1 %) had clinical symptom information available. The most commonly reported symptoms were diarrhea (89–97 % of cases depending on the phage type), abdominal pain (49–64 %), fever (43–55 %), vomiting (23–33 %), and nausea (28–36 %) (Table 2).

**Table 2:** Clinical symptoms of *Salmonella* Enteritidis cases with major phage types in Ontario, Canada, 2008–2009 (*N* = 1,123)

Phage Type		Symptom				
	Diarrhea	Bloody diarrhea	Abdominal pain	Fever	Vomiting	Nausea
	n (n/N %)	n (n/N %)	n (n/N %)	n (n/N %)	n (n/N %)	n (n/N %)
PT 1 (*N* = 114)	106 (93)	3 k	67 (59)	49 (43)	34 (30)	36 (32)
PT 4 (*N* = 63)	61 (97)	3 (5)	38 (60)	33 (52)	21 (33)	19 (30)
PT 5b (*N* = 75)	70 (93)	5 (7)	37 (49)	36 (48)	17 (23)	27 (36)
PT 8 (*N* = 333)	315 (95)	33 (10)	213 (64)	178 (54)	96 (29)	95 (29)
PT 13 (*N* = 169)	150 (89)	18 (11)	95 (56)	86 (51)	53 (31)	33 (20)
PT 13a (*N* = 178)	171 (96)	19 (11)	111 (62)	96 (54)	48 (30)	49 (28)
PT Others (*N* = 191)	180 (94)	12 (6)	117 (61)	105 (55)	59 (31)	62 (32)
All PTs (*N* = 1,123)	1,053 (94)	93 (8)	678 (60)	583 (52)	328 (29)	321 (29)

Of the 1,336 *S.* Enteritidis cases with known phage types, 1,123 cases had clinical symptom information available. *n* = number of *S.* Enteritidis cases that had the symptom. *N* = number of *S.* Enteritidis cases with the phage type. Within a row, the percentages can add up to greater than 100 % because a case could have more than one symptom

### Exposure settings

Of the 1,336 *S.* Enteritidis cases with known phage types, 372 (27.8 %) cases were missing exposure setting information, leaving 964 cases (72.2 %) available for exposure setting analysis (Table 3). International travel (19.7 % of 1,336 cases), private home (7.0 %), and food premise (6.4 %) were the most commonly reported known exposure settings. Unknown exposure setting was reported for 37.9 % of cases.

**Table 3:** Exposure settings of *Salmonella* Enteritidis cases with major phage types in Ontario, Canada, 2008–2009

Phage type	Exposure setting					
	Private home	Food premise	International travel	Other setting	Unknown	Missing
	n (n/M %)	n (n/M %)	n (n/M %)	n (n/M %)	n (n/M %)	(n/N %)
PT 1 (*N* = 132) (M = 93)	3 (3)	4 (4)	60 (65)	0	26 (28)	39 (30)
PT 4 (*N* = 76) (M = 48)	0	3 (6)	33 (69)	1 (2)	11 (23)	28 (37)
PT 5b (*N* = 83) (M = 58)	0	1 (2)	44 (76)	0	13 (22)	25 (30)
PT 8 (*N* = 398) (M = 285)	38 (13)	33 (12)	29 (10)	4 (1)	181 (64)	113 (28)
PT 13 (*N* = 198) (M = 158)	21 (13)	24 (15)	10 (6)	3 (2)	100 (64)	40 (20)
PT 13a (*N* = 218) (M = 157)	22 (14)	14 (9)	14 (9)	4 (2)	103 (66)	61 (28)
PT Others (*N* = 231) (M = 165)	9 (6)	7 (4)	73 (44)	4 (2)	72 (44)	66 (29)
Total (*N* = 1336) (M = 964)	93 (10)	86 (9)	263 (27)	16 (2)	506 (52)	372 (28)

Of the 1,336 *S.* Enteritidis cases with known phage types, 964 cases had exposure setting information available. *n* = number of *S.* Enteritidis cases with the exposure setting. M = number of *S.* Enteritidis cases that had exposure setting information available. *N* = number of *S.* Enteritidis cases with the phage type. Exposure settings in the surveillance database were categorized as: international travel (i.e., travelled outside of Canada), private home, food premise (e.g., restaurant, grocery store, bakery, deli, caterer, mobile food premise), other (e.g., institution, hospital, farm, petting zoo, child care centre), or unknown (if the only exposure reported was “unknown”)

Of the six most frequent *S.* Enteritidis phage types (*n* = 1,105), 306 (27.7 %) cases were missing exposure setting information, leaving 799 (72.3 %) cases available for exposure setting analysis. Unknown exposure setting was reported for 434 (39.3 %) of cases. Known exposure setting information was reported for 365 (33.03 %) of cases: 67 PT 1 cases (50.8 % of all PT 1 cases), 37 PT 4 cases (48.7 % of all PT 4 cases), 45 PT 5b cases (54.2 % of all PT 5b cases), 104 PT 8 cases (26.2 % of all PT 8 cases), 58 PT 13 cases (29.3 % of all PT 13 cases), and 54 PT 13a cases (24.8 % of all PT 13a cases).

Of the cases that had exposure setting information available, international travel and unknown exposure settings were the most frequently reported settings for PT 5b cases (76 and 22 % of PT 5b cases, respectively), PT 4 cases (69 and 23 % of PT 4 cases, respectively), and PT 1 cases (65 and 28 % of PT 1 cases, respectively). Unknown, private home, food premise, and international travel were the most frequently reported exposure settings for PT 8 cases (64, 13, 12, and 10 % of PT 8 cases, respectively), PT 13 cases (64, 13, 15, and 6 % of PT 13 cases, respectively), and PT 13a cases (66, 14, 9, and 9 % of PT 13a cases, respectively) (Table 3).

### Scan statistics

#### Purely spatial clusters of *S.* Enteritidis cases

**Fig. 5:**
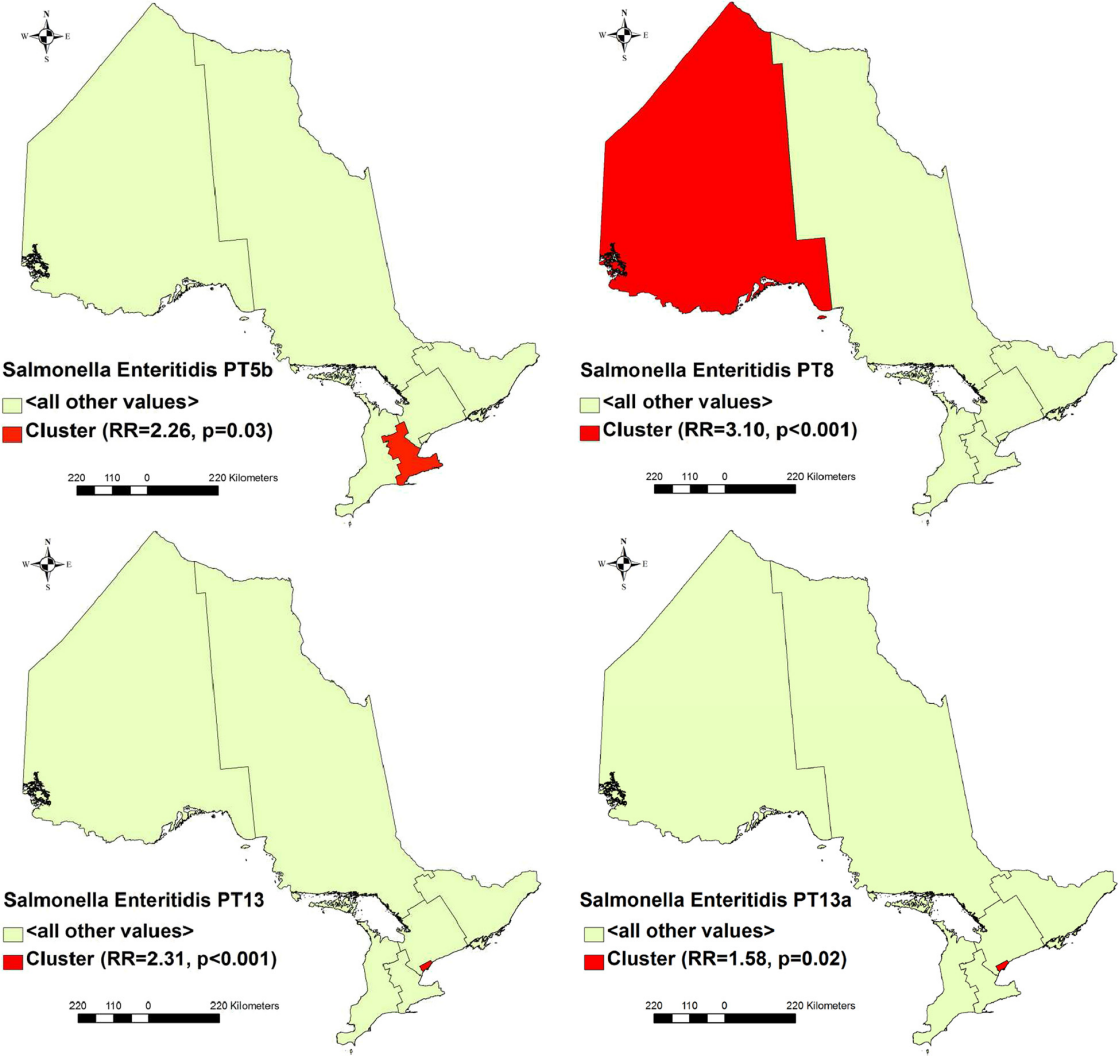
Spatial clusters of *Salmonella* Enteritidis cases with major phage types in Ontario, Canada, 2008–2009. Results based on discrete Poisson models using the SaTScan™ software. Study period: January 1, 2008 to December 31, 2009. Circular scanning window size: up to 50 % of the population at risk. Confounders controlled for: age (0–9, 10–24, 25–34, 35–49, ≥ 50 years) and sex. Criteria for reporting secondary clusters: no geographical overlap. Type of clusters investigated: high rate only. RR = relative risk. Significance level: *p* ≤ 0.05

Four significant high rate spatial clusters were detected (Table 4 and Fig. 5). A cluster of 29 PT 5b cases was identified in the Central West region (RR = 2.26, *p* = 0.003). A cluster of 22 PT 8 cases was identified in the North West region (RR = 3.10, *p* ≤ 0.001). A cluster of 74 PT 13 cases was identified in the Toronto region (RR = 2.31, *p* ≤ 0.001). A cluster of 63 PT 13a cases was identified in the Toronto region (RR = 1.58, *p* = 0.018).

**Table 4:** Clusters of *Salmonella* Enteritidis cases with the six most frequent phage types in Ontario, Canada, 2008–2009

Phage type (N)	Annual cases per 100,000	Cluster type	Region	Time frame (Year/Month)	Observed	Expected	O/E	RR	*P*-value
**PT 1 ** **(** ***N*** ** = 132)**	0.05	Space-time	North West, North East, Eastern, Central East	2008/1 to 2008/2	20	5.17	3.87	4.38	≤0.001
			Central West	2008/1 to 2008/5	16	5.24	3.05	3.33	0.046
		Temporal	All	2008/1 to 2008/3	40	16.36	2.38	3.07	0.001
**PT 4** **(** ***N*** ** = 76)**	0.03	Space-time	Eastern, Central East	2008/2 to 2008/4	15	3.89	3.85	4.55	0.010
		Temporal	All	2008/1 to 2008/11	51	34.67	1.47	2.43	0.009
**PT 5b** **(** ***N*** ** = 83)**	0.03	Spatial	Central West	NA	29	15.93	1.82	2.26	0.003
		Space-time	Central West	2008/9 to 2009/4	17	5.27	3.22	3.80	0.016
		Temporal	All	2008/12 to 2009/3	29	13.77	2.11	2.70	0.002
**PT 8 ** **(** ***N*** ** = 397)**	0.20	Spatial	North West	NA	22	7.37	2.98	3.10	≤0.001
		Space-time	North West	2009/2 to 2009/5	15	1.20	12.46	12.91	≤0.001
			South West	2008/9 to 2008/12	21	8.11	2.59	2.68	0.046
**PT 13 ** **(** ***N*** ** = 198)**	0.08	Spatial	Toronto	NA	74	40.71	1.82	2.31	≤0.001
		Space-time	Toronto	2008/4 to 2008/10	40	11.87	3.37	3.97	≤0.001
		Temporal	All	2008/4 to 2008/5	29	16.46	1.76	1.89	0.051
PT 13a (*N* = 218)	0.08	Spatial	Toronto	NA	63	44.70	1.41	1.58	0.018
		Space-time	Toronto	2009/10 to 2009/12	18	5.64	3.19	3.39	0.018
		Temporal	All	2008/7 to 2008/10	63	36.51	1.73	2.02	0.001

*N* = number of *S.* Enteritidis cases with the phage type. Results based on discrete Poisson models using the SaTScan™ software. Study period: January 1, 2008 to December 31, 2009. Time aggregation units: month. Time aggregation length: 1 month. Circular scanning window size: up to 50 % of the population at risk and/or 50 % of time the study period. Confounders controlled for: age (0–9, 10–24, 25–34, 35–49, ≥ 50 years) and sex. Criteria for reporting secondary clusters: no geographical overlap. Type of clusters investigated: high rate only. NA = not applicable. O/E = observed divided by expected. RR = relative risk. Significance level: *p* ≤ 0.05

#### Purely temporal clusters of *S.* Enteritidis cases

Five significant high rate temporal clusters were detected (Table 4). A cluster of 40 PT 1 cases occurred from January to March 2008 (RR = 3.07, *p* = 0.001). A cluster of 51 PT 4 cases occurred from January to November 2008 (RR = 2.43, *p* = 0.009). A cluster of 29 PT 5b cases occurred from December 2008 to March 2009 (RR = 2.70, *p* = 0.002). A cluster slightly above the rejection threshold of 29 PT 13 cases occurred from April to May 2008 (RR = 1.89, *p* = 0.051). A cluster of 63 PT 13a cases occurred from July to October 2008 (RR = 2.02, *p* = 0.001).

### Space-time clusters of *S.* Enteritidis cases

**Fig. 6:**
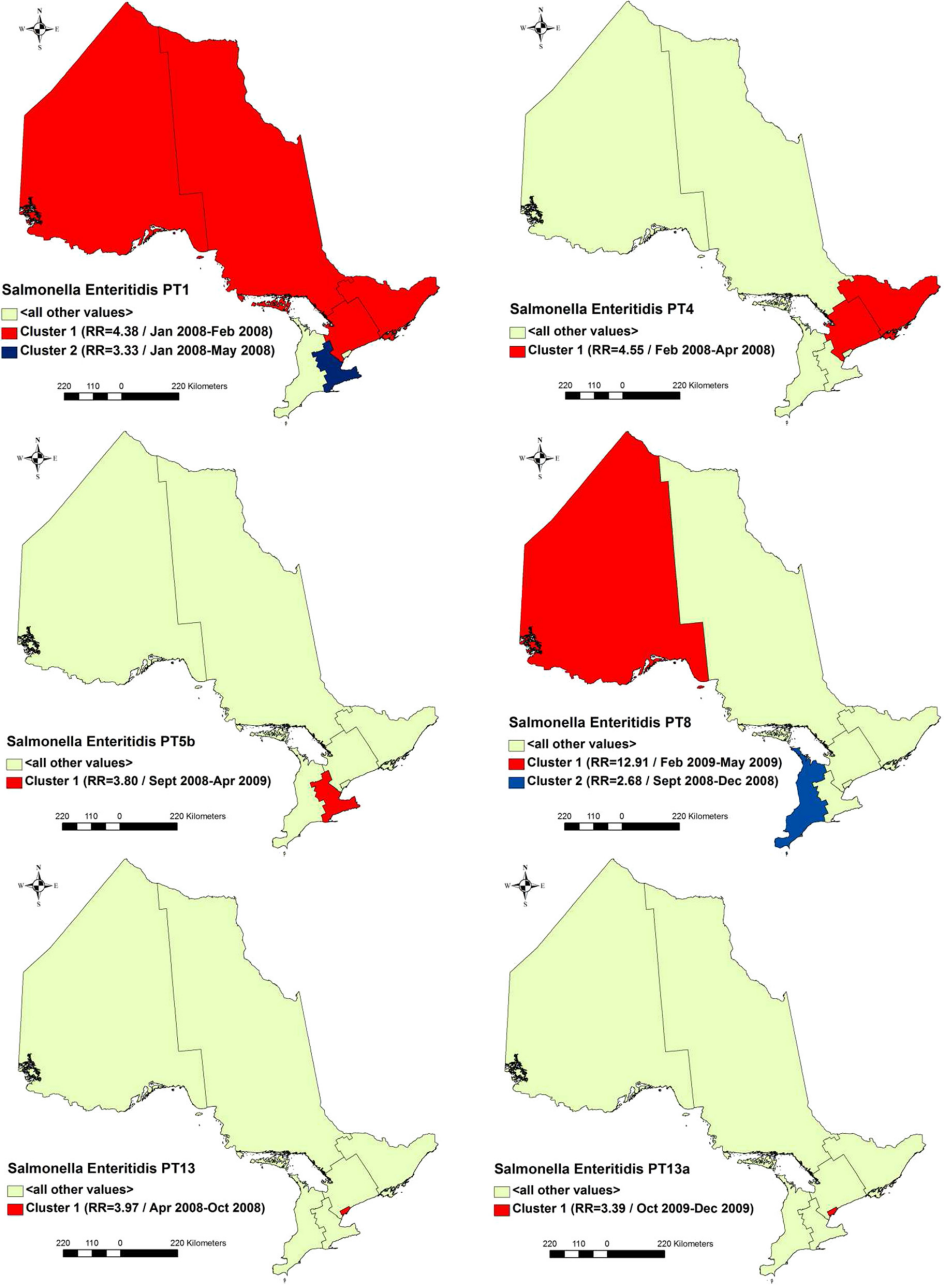
Space-time clusters of *Salmonella* Enteritidis cases with major phage types in Ontario, Canada, 2008-2009. Results based on discrete Poisson models using the SaTScan™ software. Study period: January 1, 2008 to December 31, 2009. Circular scanning window size: up to 50 % of the population at risk and 50 % of the study period. Confounders controlled for: age (0–9, 10–24, 25–34, 35–49, ≥ 50 years) and sex. Time aggregation units: month. Time aggregation length: 1 month. Criteria for reporting secondary clusters: no geographical overlap. Type of clusters investigated: high rate only. RR = relative risk. Significance level: *p* ≤ 0.05

Eight significant high rate space-time clusters were detected, including two secondary clusters (Table 4 and Fig. 6). Two clusters of PT1 cases were identified: a primary cluster of 20 cases occurred from January to February 2008 in the North West, North East, Eastern, and Central East regions (RR = 4.38, *p* ≤ 0.001); and a secondary cluster of 16 cases occurred from January to May 2008 in the Central West region (RR = 3.33, *p* = 0.046). A cluster of 15 PT 4 cases occurred from February to April 2008 in the Eastern and Central East regions (RR = 4.55, *p* = 0.010). A cluster of 17 PT 5b cases occurred from September 2008 to April 2009 in the Central West region (RR = 3.80, *p* = 0.016). Two clusters of PT 8 cases were identified: a primary cluster of 15 cases occurred from February to May 2009 in the North West region (RR = 12.91, *p* ≤ 0.001); and a secondary cluster of 21 cases occurred from September to December 2008 in the South West region (RR = 2.68, *p* = 0.046). A cluster of 40 PT 13 cases occurred from April to October 2008 in the Toronto region (RR = 3.97, *p* ≤ 0.001). A cluster of 18 PT 13a cases occurred from October to December 2009 in the Toronto region (RR = 3.39, *p* = 0.018).

### Space-time cluster cases’ exposure settings

Exposure setting information was unknown or missing for many of the cases that were part of the space-time clusters (Table 5). For the primary PT 1 cluster, exposure setting information was known for 9 of the 20 cases; seven cases reported international travel and two cases reported food premises as their exposure setting. For the secondary PT 1 cluster, exposure setting information was known for 4 of the 16 cases; all four cases reported international travel as their exposure setting. For the PT 4 cluster, exposure setting information was known for 9 of the 15 cases; all nine cases reported international travel as their exposure setting. For the PT 5b cluster, exposure setting information was known for 9 of the 17 cases; all nine cases reported international travel as their exposure setting. For the primary PT 8 cluster, no exposure setting information was known for the 15 cases. For the secondary PT 8 cluster, exposure setting information was known for 7 of the 21 cases; three cases reported food premises, two cases reported private homes, one case reported other setting, and one case reported international travel as their exposure setting. For the PT 13 cluster, exposure setting information was known for 14 of the 40 cases; 10 cases reported food premises, two cases reported private homes, one case reported other setting, and one case reported international travel as their exposure setting. For the PT 13a cluster, exposure setting information was known for 11 of the 18 cases; seven cases reported food premises, three cases reported international travel, and one case reported private home as their exposure setting.

**Table 5:** Exposure settings of the *Salmonella* Enteritidis cases included in the space-time clusters for the six most frequent phage types in Ontario, Canada, 2008-2009

Phage type Cluster			Exposure setting					
Phage type (N)	Region	Cases (n)	Private home (n)	Food premise (n)	International travel (n)	Other setting (n)	Unknown (n)	Missing (n)
PT 1 (*N* = 132)	North West, North East, Eastern, Central East	20	0	2	7	0	4	7
	Central West	16	0	0	4	0	3	9
PT 4 (*N* = 76)	Eastern, Central East	15	0	0	9	0	1	5
PT 5b (*N* = 83)	Central West	17	0	0	9	0	4	4
PT 8 (*N* = 397)	North West	15	0	0	0	0	13	2
	South West	21	2	3	1	1	5	9
PT 13 (*N* = 198)	Toronto	40	2	10	1	1	26	0
PT 13a (*N* = 218)	Toronto	18	1	7	3	0	7	0

*N* = number of *S.* Enteritidis cases with the phage type. *n* = number of *S.* Enteritidis cases with the exposure setting. Results based on discrete Poisson models using the SatTScan™ software. Study period: January 1, 2008 to December 31, 2009. Time aggregation units: month. Time aggregation length: 1 month. Circular scanning window size: up to 50 % of the population at risk and 50 % of the study period. Confounders controlled for age (0–9, 10–24, 25–34, 35–49, ≥ 50 years) and sex. Criteria for reporting secondary clusters: no geographical overlap. Type of clusters investigated: high rate only. Exposure settings in the surveillance database were categorized as: international travel (i.e., travelled outside of Canada), private home, food premise (e.g., restaurant, grocery store, bakery, deli, caterer, mobile food premise), or other (e.g., institution, hospital, farm, petting zoo, child care centre)

## Discussion

Our study enhanced the current knowledge on the spatial and temporal epidemiology of the phage types of *S.* Enteritidis that predominate in Ontario health regions. We used a step-wise approach, starting with a general exploratory analysis followed by a more specific statistical analysis. A number of phage type-specific high rate areas and time periods were identified during the exploratory analysis that were confirmed by the statistical analysis as significant spatial, temporal, or space-time clusters of cases.

Foodborne disease clusters are generally defined as the occurrence of a higher than expected number of cases for a given location and/or time period. These clusters may or may not meet the definition of an outbreak [32, 33]. Subtype-based surveillance systems frequently use the term “cluster” to describe a group of cases infected with identical microbial strains [32]. Subtyping is useful for differentiating between endemic and outbreak cases, especially for common *Salmonella* serotypes, such as Enteritidis, that occur sporadically throughout the year [34]. Differences in reservoirs and exposure settings might exist for different *S.* Enteritidis phage types, and molecular differentiation can help to understand potential sources of the different phage types [8, 34]. We defined a cluster as a health region, time period, or a health region during a particular time period with a statistically significant higher than expected phage type-specific *S.* Enteritidis infection rate. Thus, we demonstrated the effectiveness of using cluster detection tests, in conjunction with subtyping methods to understand the epidemiology of a foodborne pathogen.

A number of patterns were observed when assessing the geographical heterogeneity of health region-level IRs of *S.* Enteritidis infections for the most frequent phage types. The Central West region had the highest IRs for PTs 1, 4, and 5b, whereas the Toronto region had the highest IRs for PTs 13 and 13a. Several of these regions were later confirmed by the spatial scan statistic as regions with significant high rate clusters (e.g., cases of PT 5b significantly clustered in the Central West region and cases of PTs 13 and 13a significantly clustered in the Toronto region).

We used a smoothing method for our time-series graph to reduce the month-to-month random variation of infection rates and make the overall trends clearer. The observed trends were relatively consistent with the results of the purely temporal scan statistic, albeit not as definitive. With the exception of PT 5b, all temporal clusters occurred during 2008. Further, most clusters occurred during a distinct season. Cases of PTs 1 and 5b clustered during the winter months, cases of PT 13 clustered during the spring months, and cases of PT 13a clustered during the summer and fall months. Differences in the duration of the temporal clusters were also observed. The majority of clusters (PTs 1, 5b, 13, and 13a) were of relatively short duration (2–4 months), whereas the PT 4 cluster was of long duration (11 months). Of note, the most commonly reported phage type (PT 8) did not cluster temporally, suggesting a fairly even distribution of PT 8 cases over time throughout Ontario. A study conducted in Alberta, Canada, examining *Salmonella* serotypes rather than phage types, detected several serotype-specific temporal clusters during the 11-year study period (January 1990 to January 2002) [35]; for *S.* Enteritidis, the clusters were of short duration and occurred during the winter and spring months.

The exposure setting information is rarely confirmed by data obtained through environmental health investigations or statistical associations obtained through case-control or cohort studies [36]; however, it is considered to be useful epidemiological data for foodborne illness source attribution [37]. Knowing when, where, and why clusters occurred can aid in the development of effective outbreak detection, prevention, and control programs. Our study identified differences between phage types with respect to the time and duration of the space-time clusters, even for clusters occurring in the same region. For example, the PT 13 and 13a clusters both occurred in the Toronto region, but during different time periods (the cluster of cases with PT 13 occurred in 2008, whereas the cluster of cases with PT 13a occurred in 2009). Moreover, the cluster of cases with PT 13 was of long duration (7 months), whereas the cluster of cases with PT 13a was of short duration (3 months). Short duration clusters might signify that cases were exposed to a single infection source (e.g., point source outbreak). Long duration clusters might signify that cases were exposed to a single source (e.g., contaminated food) over a longer time period (e.g., continuous common source outbreak) [32, 33, 35], to multiple sources (e.g., continuous multiple source outbreak) [32, 33], to the occurrence of secondary infections [35], to poor food preparation practices over a prolonged period, or that the typing method used was not of high enough resolution to differentiate between different strains.

Many of the cases with PT 13 or 13a that were part of a space-time cluster reported food premises (e.g., restaurant, grocery store, bakery, deli, caterer, mobile food premise) as their main exposure setting. In North America, restaurants have been shown to be an important exposure setting for *S.* Enteritidis infections [38–41]. A number of predisposing factors for food contamination with *S.* Enteritidis in restaurants were identified, including cross contamination from raw chicken meat to food server’s hands or cutting boards due to high food volumes and food handler’s improper food safety practices during food preparation [36, 38], inadequate heat treatment of foods [38], inappropriate food storage [38], and direct contamination of food served by infected food handlers [10, 39–41]. In Ontario, *S.* Enteritidis accounted for only 10.1 % of the *Salmonella* isolates collected at pre-harvest from conventionally-raised broiler chicken flocks between July 2010 and April 2012; 65 % of the isolates were PT 13a (Tara Roberts, 2014, personal communication).

A few of the cases that were part of a PT 8, 13, or 13a space-time cluster reported private homes as their exposure setting. Previous studies identified private homes as an important exposure setting for sporadic, home-based foodborne infections [42–44]. Several predisposing factors of home-based infections have been identified, including inappropriate food handling, storage, and food preparation [42, 43]; consumption of contaminated raw and undercooked foods [42]; and person-to-person [44] and animal-to-person [45, 46] transmission.

Space-time clusters of cases with PT 1 or 4 included several overlapping health regions, occurred during nearly identical winter and spring months, and were of short duration (2-3 months). The majority of these cases reported international travel as their exposure setting. International travel was demonstrated by a number of studies as an important risk factor for *S.* Enteritidis infections in North America [15, 47, 48]. In the US, among all salmonellosis cases between 2004 and 2008, 11 % reported international travel as their exposure setting, and among those, the most commonly reported serotype was Enteritidis (22 % of travel cases) [47]. In the region of Waterloo, Ontario, Canada, between June 2005 and May 2009, 48.7 % of *S.* Enteritidis cases were international travel-related [48]. In Ontario, Canada, between July 2010 and June 2011, 51.9 % of *S.* Enteritidis infections were international travel-related, and certain phage types (e.g., 1, 4, and 5b) were isolated from cases who visited all-inclusive resorts in the Caribbean or Mexico during the winter and spring months [15]. The seasonal spike of PT 1 and 4 cases in late winter and early spring, when people often travel to warmer destinations, warrants creating advisories to inform travelers about the risks of eating abroad and how they can protect themselves against *S*. Enteritidis infections.

A number of limitations should be recognized before interpreting our study results. Surveillance programs underestimate the true burden of infections due to under-diagnosis and under-reporting of cases [3]. In Canada, it was estimated that for every reported salmonellosis case there were 26.1 unreported cases in the general population [3]. Under-reporting and under-diagnosis can be influenced by differences in populations’ medical care seeking behaviour and access to medical care [49], physicians’ specimen request and diagnosis practices [50], and laboratories testing protocols and reporting standards [50]. Regional differences in successful case follow-up should also be considered. Loss to follow-up of cases might be greater in low population density regions of the province due to difficulties encountered by public health staff in contacting cases. A large number of cases had missing or unknown exposure setting information, which might have biased our study results. The proportion and accuracy of known exposure setting information reported by investigators can depend on several factors [36], including time passed from exposure to case interview and the related recall bias, difficulty and the effort made by the investigator to contact a case, follow-up protocol and questionnaire used by the investigator (e.g., face to face interview vs. phone interview vs. questionnaire sent through the mail), a case’s willingness to be interviewed, and possible survival bias. In our study, differences in unknown exposure setting among phage types were noted. The proportion of unknown exposure setting information was higher for cases with PT 8, 13, or 13a (64–66 %) compared to cases with PT 1, 4, or 5b (22–28 %), suggesting that international travel cases had more readily available exposure history; therefore in our study, the overall proportion of cases who reported international travel as their major exposure setting was likely slightly over-estimated. Lastly, misclassification of international travel-related cases might have also occurred, especially for cases for which the incubation period was short, and for cases with a longer disease incubation period who became infected before departure [48].

Obtaining exposure setting information is a first step toward developing effective prevention and control programs; however, the location and the primary source of contamination of food products that lead to infections are not always identical [51-52]. Therefore, future research studies are needed to identify the primary source of contamination, and the type of food products that cause infections.

This study demonstrated the utility of retrospective spatial and temporal analysis of subtype-based surveillance data using exploratory and statistical methods to detect clusters of cases. Phage type-specific spatial and spatial-temporal clusters should be followed up by public health authorities to identify novel local individual-level risk factors. Increased enforcement (e.g., restaurant inspections) and education (e.g., food safety training for restaurant employees and the general public) in health regions with identified spatial or spatial-temporal clusters have the potential to decrease the incidence of PTs 8, 13, and 13a. Further, prevention programs (e.g., travel advisories) that are targeted during the winter and spring months have the potential to decrease the incidence of PTs 1, 4, and 5b. During the study period no outbreaks were reported in Ontario; thus, the evaluation of current outbreak detection methods used by public health staff at various PHUs is warranted. Future studies are needed to evaluate the frequency of false positive clusters, to assess the effectiveness of cluster detection using statistical methods, to compare the more traditional outbreak investigation procedures to scan statistic cluster detection techniques, and to measure the feasibility of statistical methods for identifying infection clusters. Purely spatial or purely temporal clusters might be the result of a space-time cluster, which should be considered when evaluating our study results. There is a need also for prospective research studies to identify clusters of *S*. Enteritidis infections in real-time (e.g., weeks, months), and to assess and evaluate individual-level risk factors for infections included in these clusters. Moreover, there is a need for high resolution molecular subtyping methods (e.g., multiple locus variable-number tandem repeat analysis or whole genome sequencing) to better understand relationships between cases in a cluster.

## Conclusions

This is the first study that has evaluated the spatial and temporal epidemiology of the phage types of *S.* Enteritidis that predominate in Ontario health regions. This study demonstrated the value of using a number of spatial-temporal and subtyping methods to better understand the epidemiology of a foodborne illness, such as salmonellosis. Our study highlighted phage type-specific differences in spatial distributions, temporal trends, clinical symptoms, exposure settings, and space-time clusters of *S.* Enteritidis infections. Several health regions were identified with increased phage type-specific *S.* Enteritidis infection rates where future studies should be conducted to identify novel individual-level risk factors, and where future prevention and control programs should be targeted to reduce the incidence of *S.* Enteritidis infections. Our study methodology may be applicable to other foodborne disease surveillance data.

## Additional file

Additional file 1:
**Legend 1 for Fig.** **1** **.** Ontario Public Health Unit labels, names, and population estimates. (DOCX 16 kb)

## Abbreviations

GIS: Geographic information system
iPHIS: Integrated Public Health Information System
IR: Incidence rate
MOHLTC: Ontario Ministry of Health and Long-Term Care
PHU: Public health unit
PT: Phage type
*S.* Enteritidis: *Salmonella enterica* serovar Enteritidis
US: United States of America
