# Supplementary material for: Spatial-temporal epidemiology of human Salmonella Enteritidis infections with major phage types (PTs 1, 4, 5b, 8, 13, and 13a) in Ontario, Canada, 2008–2009
Source: BMC Public Health. 2015 Dec 17;15:1247. doi: 10.1186/s12889-015-2592-6 (PMC4682253; doi:10.1186/s12889-015-2592-6)
Supplement: Additional file 1: — Legend 1 for Fig. 1 . Ontario Public Health Unit labels, names, and population estimates. (DOCX 16 kb) [file 12889_2015_2592_MOESM1_ESM.docx]

| **Label** | **Public Health Unit** | **Health Region** | **Population** |
| --- | --- | --- | --- |
| 0 | The District of Algoma Health Unit | North East | 118,594 |
| 1 | Brant County Health Unit | Central West | 138,538 |
| 2 | Durham Regional Health Unit | Central East | 612,247 |
| 3 | Elgin-St. Thomas Health Unit | South West | 90,074 |
| 4 | Grey Bruce Health Unit | South West | 163,416 |
| 5 | Haldimand-Norfolk Health Unit | Central West | 111,823 |
| 6 | Haliburton, Kawartha, Pine Ridge District Health Unit | Central East | 177,736 |
| 7 | Halton Regional Health Unit | Central West | 493,450 |
| 8 | City of Hamilton Health Unit | Central West | 530,785 |
| 9 | Hastings and Prince Edward Counties Health Unit | Eastern | 162,777 |
| 10 | Huron County Health Unit | South West | 60,921 |
| 11 | Chatham-Kent Health Unit | South West | 110,458 |
| 12 | Kingston, Frontenac, and Lennox and Addington Health Unit | Eastern | 194,212 |
| 13 | Lambton Health Unit | South West | 132,173 |
| 14 | Leeds, Grenville and Lanark District Health Unit | Eastern | 168,219 |
| 15 | Middlesex-London Health Unit | South West | 451,884 |
| 16 | Niagara Regional Area Health Unit | Central West | 443,064 |
| 17 | North Bay Parry Sound District Health Unit | North East | 126,394 |
| 18 | Northwestern Health Unit | North West | 83,548 |
| 19 | City of Ottawa Health Unit | Eastern | 881,833 |
| 20 | Oxford County Health Unit | South West | 106,898 |
| 21 | Peel Regional Health Unit | Central East | 1,308,538 |
| 22 | Perth District Health Unit | South West | 77,228 |
| 23 | Peterborough County-City Health Unit | Central East | 138,516 |
| 24 | Porcupine Health Unit | North East | 86,717 |
| 25 | Renfrew County and District Health Unit | Eastern | 103,588 |
| 26 | The Eastern Ontario Health Unit | Eastern | 199,134 |
| 27 | Simcoe Muskoka District Health Unit | Central East | 513,367 |
| 28 | Sudbury and District Health Unit | North East | 200,041 |
| 29 | Thunder Bay District Health Unit | North West | 157,100 |
| 30 | Timiskaming Health Unit | North East | 34,593 |
| 31 | Waterloo Health Unit | Central West | 517,090 |
| 32 | Wellington-Dufferin-Guelph Health Unit | Central West | 272,091 |
| 33 | Windsor-Essex County Health Unit | South West | 402,663 |
| 34 | York Regional Health Unit | Central East | 1,013,932 |
| 35 | City of Toronto Health Unit | Toronto | 2,685,203 |
